# Chloride intracellular channel 1 (CLIC1) contributes to modulation of cyclic AMP‐activated whole‐cell chloride currents in human bronchial epithelial cells

**DOI:** 10.14814/phy2.13508

**Published:** 2018-01-25

**Authors:** Bo Liu, Charlotte K. Billington, Amanda P. Henry, Sangita K. Bhaker, Alexander K. Kheirallah, Caroline Swan, Ian P. Hall

**Affiliations:** ^1^ Division of Respiratory Medicine The University of Nottingham Nottingham United Kingdom

**Keywords:** Chloride channels, CLIC1, cyclic AMP, human bronchial epithelial cells

## Abstract

Chloride channels are known to play critical physiological roles in many cell types. Here, we describe the expression of anion channels using RNA Seq in primary cultures of human bronchial epithelial cells (hBECs). Chloride intracellular channel (CLIC) family members were the most abundant chloride channel transcripts, and CLIC1 showed the highest level of expression. In addition, we characterize the chloride currents in hBECs and determine how inhibition of CLIC1 via pharmacological and molecular approaches impacts these. We demonstrate that CLIC1 is able to modulate cyclic AMP‐induced chloride currents and suggest that CLIC1 modulation could be important for chloride homeostasis in this cell type.

## Introduction

Chloride (Cl^−^) channels are critically important in many physiological processes including muscle contraction, neuronal excitation, cell‐volume regulation, transepithelial fluid transportation, acidification of intracellular organelles, and mucus production and secretion (Edwards and Kahl 2010). Dysfunction of Cl^−^ channels has been implicated in many diseases including osteoporosis, epilepsy, and myotonia with one of the most intensively investigated diseases being cystic fibrosis (CF) (Verkman 2009). CF is caused by mutations of the cystic fibrosis transmembrane conductance regulator (CFTR), a cyclic AMP (cAMP)‐activated Cl^−^ channel expressed in the apical membrane of epithelial cells in airways, intestine, pancreas, testis, sweat ducts, and other fluid transporting tissues (Haq et al. 2016). However, investigations using bronchial epithelial cells isolated from patients with CF revealed not only decreased CFTR activity following stimulation of cells with cAMP, but also changes in other Cl^−^ channels such as Ca^2+^‐activated Cl Channels (CACC), Outwardly rectified Cl Channels (ORCC) and volume‐activated Cl Channels (VACC), which were all activated to some extent by an increase in cell cAMP (Cliff and Frizzell 1990; Anderson and Welsh 1991; Schwiebert et al. 1995). An extensive set of proteins are known to interact with CFTR (Wang et al. 2006), and it is likely that these changes are related to modulation of other Cl^−^ channels either because of direct binding to CFTR or because of compensatory changes which occur due to defective CFTR function. Altered activity of these other Cl^−^ channels are of interest as targeting these could provide an alternative therapeutic strategy in cystic fibrosis.

Of the multiple Cl^−^ channels present in human bronchial epithelial cell (hBEC)s, the potential role of the chloride intracellular channel (CLIC) family of proteins has not been studied. There are seven family members (CLIC1, CLIC2, CLIC3, CLIC4, CLIC5, p64, and parchorin) and, through the course of this study, we identified CLIC1 as the member most highly expressed in airway epithelial cells. Hence, we sought to determine the function of CLIC1 in hBECs in more depth. The aims of this study were therefore to characterize the expression profile of CLIC family members in hBECs, and to investigate the potential contribution of CLIC1 to modulation of Cl^−^ channel activity in these cells.

## Materials and Methods

### Human bronchial epithelial cells

hBECs (Lonza, Wokingham, UK) were cultured and passaged in growth factor‐supplemented medium (BEGM, Lonza) as per manufacturer's instructions. Cells were used between passage 2 and 4 for all experiments. Three donors were used.

### Quantification of CLIC homologs in hBEC by RNA sequencing

Total RNA was extracted from hBECs from three donors (Lonza, UK) at either passage 3 or 4 using the GenElute Mammalian Total RNA Miniprep Kit (Sigma‐Aldrich) or the RNeasy RNA extraction kit (Qiagen). Donor 1 was a Caucasian, 56‐year‐old male smoker; Donor 2 was a 19‐year‐old male smoker, and Donor 3 was a Caucasian, 50‐year‐old male smoker. RNA quality was assessed using the Agilent 2100 Bioanalyzer with all samples having a RNA integrity number ≥ 8. A sequencing library was prepared using the Next Ultra Directional RNA Library Prep kit for Illumina (New England Biolabs). mRNA was poly‐A selected using Dynabeads Oligo (dT)25 (Life technologies). cDNA was synthesized using random primers. Finally, a cDNA library was prepared by end‐repair, phosphorylation, A‐tailing, adapter ligation, and PCR amplification. Paired‐end sequencing was performed on Illumina NextSeq500 with approximately 40 million reads per sample generated. The raw read FastQ files (75 base pair reads) were quality evaluated using FastQC. Reads were used for subsequent analysis using the Ubuntu 12.04 LTS operating system. Reads were aligned to the human genome (Build GRCh37) for each sample using Bowtie2 tool as part of TopHat (v2.0.12). The Cufflinks v2.2.1 program was used to assemble transcriptomes for each individual sample. Transcriptomes from all the samples were merged by reference annotation based transcript assembly (RABTA) using Cuffmerge v1.0.0 to provide the maximum reads to identify low‐expression transcripts and mean fragments per kilobase of transcript per million fragments mapped (FPKM) expression was quantified using Cuffnorm v2.2.1.

### Splice variant analysis of CLIC homologs in hBEC

Total RNA was extracted from hBECs from two donors (Lonza, UK) at either passage 3 or 4 using the GenElute Mammalian Total RNA Miniprep Kit (Sigma‐Aldrich). Donor 1 was a 19‐year‐old male smoker and Donor 2 was a Caucasian, 50‐year‐old male smoker. RNA sequencing and data analysis was performed as described ((Kheirallah et al. 2017)15). The Cuffmerge generated novel gene transfer format (GTF) annotation file was compared to the Ensembl GTF annotation of the GRCh37 genome build using Cuffcompare v2.2.1. Basal hBEC sample data (*n* = 2) were used to determine splice variants. Splicing graphs were generated using SpliceGrapher v0.2.4.

### Whole‐cell patch clamp investigations of CLIC1 currents

Cultured hBECs were trypsinized and dispersed in a Petri dish coated with 0.1% l‐Lysine solution and perfused constantly by gravity with extracellular solution (PSS; NaCl 140 mmol/L, CsCl 5 mmol/L, Ca_2_Cl 1 mmol/L, Mg_2_Cl 1 mmol/L, HEPES 10 mmol/L, and glucose 10 mmol/L, pH 7.4). A whole‐cell patch clamp technique was used to study CLIC1 currents specifically utilizing an EPC10 Double amplifier (HEKA, Germany) and Patchmaster (version 2 × 73.5, HEKA, Germany). Borosilicate pipettes were pulled via an electrode puller (PC‐10, Narishige, Japan) with a resistance of 3–5 mΩ when filled with the electrode solution (CsCl 140 mmol/L, EGTA 5 mmol/L, MgCl_2_ 1 mmol/L, CaCl_2_ 1 mmol/L, HEPES 10 mmol/L, glucose 10 mmol/L, pH 7.4 adjusted with cesium hydroxide). The free Ca^2+^ concentration for this electrode solution was 14.26 nmol/L using a Ca^2+^‐EGTA calculator (V 1.2, Maxchelator). Cells were voltage‐clamped from a holding potential of −40 mV and current–voltage relationships analyzed by applying voltage ramps (from −100 mV to 100 mV) at a rate of 0.5 Hz. Currents were sampled at 5 kHz, digitally filtered at 1 kHz, and stored on disc for future analysis. The calculated voltage error due to series resistance was always <5 mV. Capacitance and series resistance readings were continuously monitored and the recording was discarded if a significant change (>25%) occurred in either parameter. The outward currents (mean currents at 100 mV) and the inward currents (mean currents at ‐100 mV) were taken from steady‐state (when three consecutive recordings exhibited identical late and instantaneous current amplitudes) I/V curves and plotted against time. Experimental drugs were prepared as stock solutions then further diluted in extracellular solution to reach the desired final concentrations. These were perfused through a pressurized puffer pipette positioned near the cells: a separate pressurized pipette was used to wash out drugs (modified DAD‐8 Macro Manifold perfusion system, reported time for switching between solutions ~12 msec). When DMSO was used to dissolve experimental drugs, the same amount of DMSO was added into the control external solution. The final DMSO concentration used was always less than 0.1%, which had no significant effects on whole‐cell currents (data not shown).

For determination of anion permeability, Cl^−^ in both internal and external solutions was replaced with the same amount of different anions (I^−^, Br^−^, or Aspartate^−^). Amplitudes of the currents and reversing potentials were calculated using the analysis software Patchmaster based on the Goldman–Hodgkin–Katz equation. For all these experiments, an agar salt bridge (0.5% Agar in 3mol/L KCl) was used to connect the Ag/AgCl electrode. Junction potentials were measured using Patchmaster (version 2 × 73.5, HEKA, Germany) to correct the results.

### siRNA knockdown experiments targeted against CLIC1

hBECs (passage 2) were seeded into a six‐well plate at a density of 120,000 cells per well then allowed to adhere and proliferate for 24 h before transfection. Initial experiments determined which of three unique 27mer siRNA duplex siRNAs designed to target CLIC1 (Origene UK) were most effective. A single siRNA was selected for the remaining experiments and was transfected into hBECs using the INTERFERin^®^ transfection reagent (Polyplus, at a final concentration of 2 *μ*L/mL) as per manufacturer's instructions. For these studies, siRNA was added into the wells to give a final concentration of 1 nmol/L. Following transfections, cells were incubated for 48 h before being harvested for patch clamp studies, RNA or protein extraction for subsequent analysis by RT‐PCR or Western blot as described below.

### RT‐PCR analysis of CLIC1 expression

Cultured cells were lysed, RNA extracted (RNeasy mini kit, Qiagen, Crawley, UK), and cDNA synthesized with Superscript II (Invitrogen, Paisley, UK) as outlined in the manufacturers’ protocols. A predesigned TaqMan Gene Expression Assay (Applied Biosystems, (Bleiswijk, Netherlands) was selected which amplifies a 74 bp region spanning exons 1 and 2 as this region was found to be conserved throughout all isoforms of CLIC1 described in the literature and, specifically, those isoforms we had detected in hBECs via RNA sequencing (described in results).

### Analysis of CLIC1 protein expression via western blotting

Forty‐eight hours following transfection, hBECs were washed three times in cold PBS then 50 *μ*L lysis buffer was added per well (lysis buffer: 1 mL of unsupplemented Cytobuster™ lysis buffer with Benzonase Nuclease 1 *μ*L/mL, DTT 1 mmol/L, Pepstatin A 1 *μ*g/mL). Cells were scraped from the surface of the culture vessel plastic and lysates incubated on ice for 30 min prior to being centrifuged at 15115 *g* for 10 min at 4°C to pellet insoluble material. A quantity of 40 *μ*g protein per experimental condition (control, scrambled and siRNA against CLIC1 treated) was mixed with SDS loading buffer and subjected to SDS‐polyacrylamide gel electrophoresis on a 10% polyacrylamide gel and electrophoretically transferred to a PVDF membrane. A monoclonal antibody against full length recombinant protein corresponding to human CLIC1 (Clone CPTC‐CLIC‐1, Cat. # MABN46, Merck Millipore) was diluted at a final concentration of 1 *μ*g/mL, and incubated with the membrane at 4°C overnight, to identify CLIC1 protein on the membrane. Following treatment of the membrane with goat‐anti‐mouse immunoglobulin G coupled to peroxidase (1:5000), staining was performed with the enhanced chemoluminescence (ECL) western blotting analysis system. The same filter membrane was then stripped of antigens with western blotting stripping buffer (21059, Thermo Scientific) allowing subsequent detection of the expression of the house‐keeping protein, *β‐*actin, which was picked up by a polyclonal antibody at a concentration of 1 *μ*g/mL and a goat‐anti‐rabbit secondary antibody (0.25 *μ*g/mL) conjugated to peroxidase and detected by use of the same ECL western blotting system.

### Statistical analysis

All summary data are shown as mean ± SE mean. Statistical significance between groups was assessed by unpaired t tests where *P < 0.05, **P < 0.01 were considered significant unless otherwise stated. An one‐way ANOVA with Bonferroni's post hoc test was used to analyze RT‐PCR results.

## Results

### Identification of CLIC homologs in hBECs

RNAseq data from six separate experiments from which RNA Seq had been isolated from hBEC cultures under basal (unstimulated) conditions were used to identify anion channel transcripts present in hBECs. CLIC channels were found to be expressed most highly accounting for 61%, with the next most highly expressed channel family being volume‐regulated anion channels (18%, see Table S1A). Expression was observed for six CLIC family members; however, CLIC2, 3, 5, and 6 were only expressed at very low levels. When the abundance of CLIC1 and four was compared, 80% of all transcripts were CLIC1 thus further investigations focused on this subfamily (Table S1B). Five different transcripts of CLIC1 were identified, four of which have previously been reported (CLIC1‐001, ‐002, ‐003, and ‐004 also known as ENST00000375784, ENST00000375780, ENST00000395892 and ENST00000375779, respectively). The novel isoform exhibited a modified 5′ end comprising of the furthest 5′ exon of CLIC1‐004, followed by an exon which occurs second in CLIC1‐002 and third in CLIC1‐003. Downstream of this, all five variants are identical.

Given this clear evidence for the expression of CLIC homologs in hBECs, we next set out to examine whether or not the most abundant isoform (CLIC1) contributes to the control of chloride homeostasis in these cells. Initially, we characterized chloride currents in hBECs and then explored inhibition of CLIC1 to determine the potential contributions of CLIC1 specifically to the overall currents. Interestingly, no transcript for CFTR was found in any of the donors studied.

### Anion permeability of hBECs

**Figure 1:**
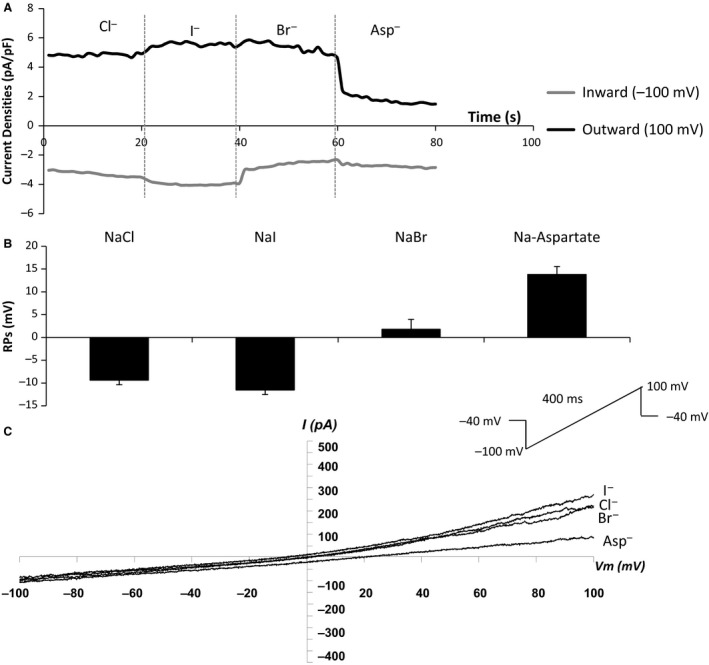
*Anion permeation study demonstrates the relative permeability of four anions (I* ^*−*^ *> Cl* ^*−*^ *> Br* ^*−*^ *> Na* ^*+*^ *> aspartate* ^*−*^ *) in hBECs*. A: Replacement of Cl^−^ with I^−^, Br^−^ or aspartate caused a reduction of the outward currents while reversing potentials moved positively; B: Corresponding changes of reversing potentials; C: I/V curves of anion currents in hBEC cells. *n* = 5 for all panels.

Initial experiments sought to determine the anion permeability of the hBECs. When the cells were hyperpolarized from −40 mV to −100 mV and then depolarized to 100 mV and symmetrical Cl^−^ was used as a main anion, the reversing potential was −9.38 ± 0.65 mV (average from five cells). When Cl^−^ was replaced with I^−^, reversing potentials became more negative (−11.54 ± 1.64 mV, *n* = 5) while replacing with Br^−^ displaced reversing potentials more positively (1.78 ± 2.77 mV, *n* = 5). Aspartate^−^ changed reversing potentials to 13.80 ± 2.74 mV (*n* = 5) with a greater inhibition of the outward currents induced (Fig. 1). This suggests that the anion permeability of the cells under the described conditions was I^−^>Cl^−^>Br^−^>aspartate^−^.

### Effects of isoproterenol and forskolin on Cl^−^ currents

**Figure 2:**
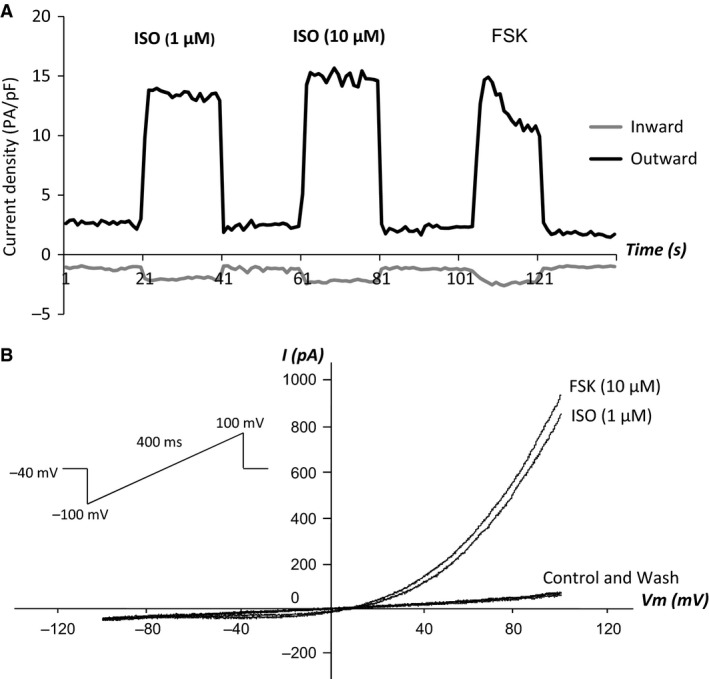
*Inducers of cyclic AMP increase Cl* ^*−*^ *currents in hBEC cells* A: Effects of perfusion of the *β‐*adrenoceptor agonist, isoproterenol (ISO) (1 *μ*mol/L then 10 *μ*mol/L) or the direct activator of adenylyl cyclase, forskolin (FSK) (10 *μ*mol/L) on Cl^−^ currents in hBEC cells; B: I/V curves of corresponding currents. *n* = 6 for both panels.

Exposure of hBECs to the *β‐*adrenoceptor (*β*AR) agonist isoproterenol (1 or 10 *μ*mol/L) or the direct activator of adenylyl cyclase (AC), forskolin (10 *μ*mol/L) induced a significant increase in outwardly rectifying currents with hardly any change observed to inward currents (an increase from 1.43 ± 0.3 to 22.69 ± 0.69 pA/F, *n* = 6, *P* < 0.01). The currents were readily reversible following wash out of drug. No “rundown” phenomenon was observed in the 40 min’ recording time, despite the absence of ATP from the electrode solution (Fig. 2).

### Identity of the currents induced by isoproterenol and forskolin

A range of Cl^−^ blockers known to modulate CLIC channels was chosen and their effect on the whole‐cell currents induced by isoproterenol (1 *μ*mol/L) investigated. The agents used and a brief description are given herewith. Phloretin is a bisphenol derivative which preferentially blocks volume‐sensitive Cl^−^ channels and cyclic AMP‐Cl^−^ channels at higher concentrations. The aromatic compound, 9‐anthracenecarboxylic acid (9‐AC) is a Cl^−^ transport inhibitor shown to have a moderate to strong inhibitory action on PKA‐activated cardiac Cl^−^ channels. 5‐Nitro‐2‐(3‐phenylpropylamino) benzoic acid (NPPB), 4, 4′‐diisothiocyanato‐2,2′‐stilbenedisulfonic acid disodium salt (DIDS), and niflumic acid (NAC) are Cl^−^ channel blockers, with known selectivity for Ca^2+^‐activated Cl^−^ channels. DIDS, NPPS, and phloretin have all been shown to inhibit CLIC channels in primary cardiac myocytes (Malekova et al. 2007). Finally, indanyloxyacetic acid 94, (IAA‐94) was used as a partially selective inhibitor of CLIC channels (Edwards 2006).

**Figure 3:**
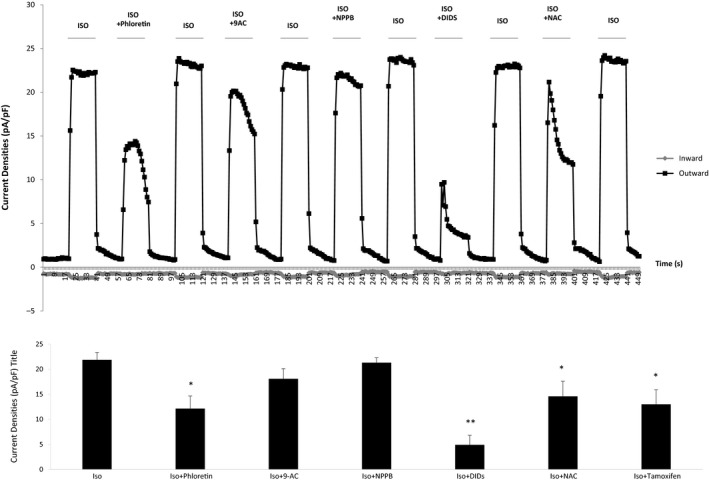
*Effect of a range of Cl* ^*−*^ *channel blockers on outwardly rectifying Cl* ^*−*^ *currents induced by isoproterenol in hBEC cells*. Cells were perfused with isoproterenol (1 *μ*mol/L) alone or in combination with a range of reported Cl^−^ channel blockers namely 2′,4′,6′‐trihydroxy‐3‐(4‐hydroxyphenyl) propiophenone (P, 20 *μ*mol/L, *n* = 9), 9‐anthracenecarboxylic acid (9‐AC, 50 *μ*mol/L, *n* = 9), 5‐nitro‐2‐(3‐phenylpropylamino)benzoic acid (NPPB, 50 *μ*mol/L, *n* = 9), 4,4′‐diisothiocyanato‐2,2′‐stilbenedisulfonic acid disodium salt (DIDS, 50 *μ*mol/L, *n* = 9), niflumic Acid (NAC, 50 *μ*mol/L, *n* = 9). W denotes washout period.

In these studies, DIDS (50 *μ*mol/L) significantly reduced isoproterenol‐induced whole‐cell currents by 79% (23.49 ± 0.7 vs. 4.92 ± 1.89 pF/pA, *P* < 0.01, *n* = 9). Phloretin (20 *μ*mol/L) reduced the currents by 44.5% (21.87 ± 1.48 vs. 12.14 ± 1.52 pF/pA, *P* < 0.01, *n* = 9) and NAC (50 *μ*mol/L) reduced by 35.5% (22.59 ± 1.51 vs. 14.58 ± 3.0 pF/pA, *P* < 0.01, *n* = 9). NPPB (50 *μ*mol/L) did not alter whole‐cell currents induced by isoproterenol (22.8 ± 0.61 vs. 22.3 ± 1.0 pA/pF, *P* > 0.05, *n* = 9) (Fig. 3).

**Figure 4:**
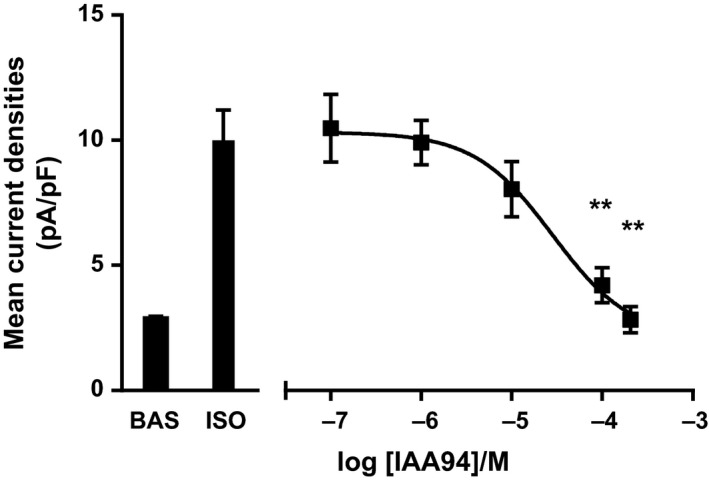
*IAA‐94 inhibits isoproterenol‐induced Cl* ^*−*^ *currents in a dose‐dependent manner*. Perfusion of hBECs with a range of doses of the CLIC1 blocker IAA‐94, inhibited 1 *μ*mol/L isoproterenol‐induced Cl^−^ Channel activation in a dose‐dependent manner (*n* = 10, ** *P* < 0.01 compared with response to isoproterenol alone).

The chloride ion channel inhibitor (IAA‐94) inhibited the whole‐cell currents induced by isoproterenol in a dose‐dependent manner and complete inhibition was achieved following exposure to 200 *μ*mol/L (Fig. 4).

### CFTR antagonists inhibit Cl^−^ currents induced by forskolin

**Figure 5:**
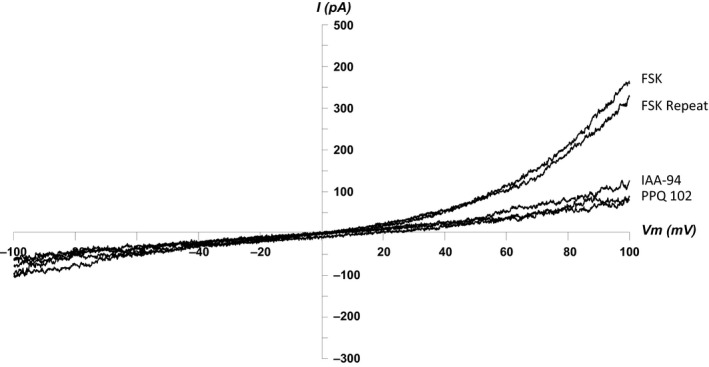
: *CFTR Blockers abolish forskolin‐induced Cl* ^*−*^ *currents in hBECs*. I/V curve showing that PPQ 102 (180 nmol/L) and IAA‐94 (100 *μ*mol/L) abolish Cl^−^ currents induced by forskolin (10 *μ*mol/L) in hBEC cells (*n* = 8).

7,9‐Dimethyl‐11‐phenyl‐6‐(5‐methylfuran‐2‐yl)‐5,6‐dihydro‐pyrimido[4′,5′‐3,4]pyrrolo[1,2‐a]quinoxaline‐8,10(7*H*,9*H*)‐dione, PPQ‐102 has been reported as being one of the most potent and specific inhibitors of CFTR currents available, with an IC_50_ of ~90 nmol/L. PPQ‐102 (180 nmol/L) abolished the majority of whole‐cells currents induced by forskolin to a comparable degree to that observed following exposure to IAA‐94 (*n* = 8) (Fig. 5). Interestingly, interrogation of our RNA Seq dataset found no evidence of CFTR transcript in any of the donors used. While it is possible that some protein could be present, it seems more likely that this CFTR blocker has off‐target effects which require additional investigation.

### siRNA‐mediated knockdown of CLIC1 mRNA and protein reduces forskolin‐induced Cl‐ currents

The above data demonstrate that there is a cAMP‐stimulated Cl^−^ current in hBECs but also suggest that inhibition of CLIC1 can modulate cAMP‐stimulated Cl^−^ currents either directly or indirectly. Given the problems with lack of specificity of many of the antagonists available to study Cl^−^ channel function, we further explored the role of CLIC in modulating these currents by utilizing siRNA specifically targeted against CLIC1.

**Figure 6:**
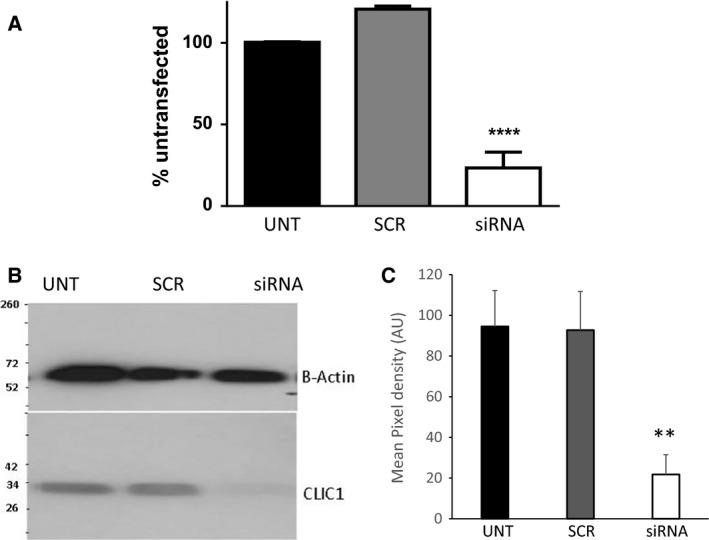
*Transfection of siRNA specifically targeted against CLIC1 significantly reduces both mRNA and protein expressions of CLIC1 in cultured hBEC cells*. Forty‐eight hours posttransfection of CLIC1‐targeted siRNA (1 nmol/L) both mRNA (duplicate *n* = 3) (A) and protein (B and C) expression of CLIC1 was reduced in cultured hBEC cells (B is a representative image of *n* = 6, **** denotes *P* < 0.0001 and ** denotes *P* < 0.001 compared with scrambled control). Western blots were stripped and reprobed for *β*‐actin. mRNA expression is normalized to housekeeper via the 2^−ΔΔCT^ method.

Transfection of the cells with siRNA specifically targeted against CLIC1 at a concentration of 1 nmol/L significantly reduced mRNA expression of CLIC1 by 76.7 ± 9.7% (three separate experiments performed in duplicate, *P* < 0.0001), Figure 6A. The corresponding protein expression of CLIC1 in matched samples was also markedly reduced as assessed via Western blot (Fig. 6B).

**Figure 7:**
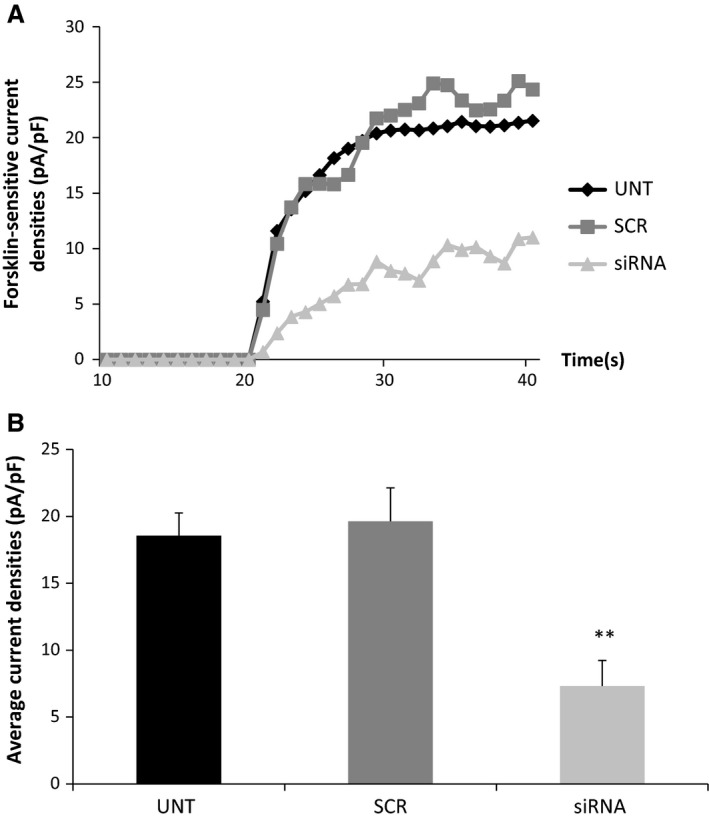
*siRNA‐mediated knockdown of CLIC1 significantly inhibits forskolin‐induced Cl* ^*−*^ *currents* Effect of transfection of siRNA targeting CLIC1 in hBECs on forskolin (10 *μ*mol/L)‐sensitive Cl^−^ currents shown as a representative trace (A) and averages of the forskolin‐sensitive currents in untransfected (*n* = 23), scrambled (*n* = 18) and siRNA group (*n* = 18) were compared (B).

Having demonstrated successful knockdown of CLIC1 at both mRNA and protein levels, the effects of inhibition of CLIC1 expression on the basal whole‐cell currents and those induced by forskolin were investigated. The different experimental groups (untransfected, scrambled siRNA, and siRNA targeting CLIC1) were studied blinded to treatment. In untransfected cells forskolin (10 *μ*mol/L) induced an average increase of the whole‐cell current densities of 18.56 ± 1.7 pA/pF (*n* = 23) and in the scrambled siRNA group an increase of 19.64 ± 2.5 (*n* = 18). However, in cells transfected with siRNA targeting CLIC1 the increased whole‐cell current densities were observed to significantly decrease by 62.7% (c.f. scrambled control, *n* = 18, *P* < 0.01) (Fig. 7). There was no effect of CLIC1 inhibition on basal current when compared with scrambled control or untransfected.

## Discussion

In vivo, human airway epithelial cells express a range of chloride channels which can be induced by cAMP including CFTR, outwardly rectifying Cl^−^ channels, Ca^2+^‐activated Cl^−^ channels and volume‐activated Cl^−^ channels (Cliff and Frizzell 1990; Anderson and Welsh 1991; Schwiebert et al. 1995). While the differentiated human airway epithelium contains mucus secreting cells, ciliated epithelial cells, and basal cells, CFTR is predominantly expressed in the apical epithelium. Here, we studied the expression of Cl^−^ channels in hBECS, which are representative of the basal epithelial cell layer, and which play an important role in maintaining epithelial homeostasis (Wadsworth et al. 2006). In this study, we report that a number of Cl^−^ channels are also expressed in hBECs and provide evidence using two different approaches that CLIC family members are relatively abundant, and specifically that CLIC1 is able to modulate cAMP‐induced chloride currents.

The majority of interest in CLIC1 to date has focused on its role in phagosome acidification and proteolysis, which in inflammatory cells makes CLIC1 a potential target for anti‐inflammatory drugs (Salao et al. 2016). CLIC1 is believed to be able to cycle between membrane‐inserted and soluble forms (Tulk et al. 2002). Current suggestions are that CLIC proteins act as second messengers which can translocate to the membrane in response to oxidative stress and this translocation can be transient or chronic (Peretti et al. 1848). Thus, CLIC1 has been identified as a sensor of cell oxidative stress (Averaimo et al. 2010). CLIC1 translocation has also been observed to be pH‐dependent. CLIC1 is overexpressed in a number of malignant tumors and this may play a role in tumor invasion and metastasis (Peretti et al. 1848), and in addition CLIC1 has been shown to be capable of promoting neurite outgrowth when stimulated by protein kinase A through an undefined pathway (CLIC1 knockout mice exhibit platelet dysfunction and inhibited clotting) (Qiu et al. 2010). However, CLIC1 may also act at the cell membrane as a Cl^−^ channel, mediating Cl^−^ flow and thus water transportation. Purified CLIC1 protein has also been observed to form functional channels capable of conducting Cl^−^ currents when reconstituted in lipid bilayer membranes or expressed in CHO cells (Tonini et al. 2000; Warton et al. 2002). CLIC1 is known to be expressed in murine bronchial epithelia (Ulmasov et al. 2007).

In hBECs, we observed whole‐cell anion currents to be induced by the nonselective *β*‐adrenoceptor agonist isoproterenol and the direct activator of AC forskolin. These are unlikely to be mostly due to a contribution from Ca^2+^‐activated Cl^−^ channels, as our experimental conditions were specifically designed to minimize the contribution of these channels, with the free Ca^2+^ in the electrode solution being only 14 nmol/L, which is far below the micromolar concentration of free Ca^2+^ reported to activate these channels. However, there may be a small residual contribution of these channels as NAC was partially able to inhibit the whole‐cell currents induced. Similarly, these currents are also unlikely to be due to volume‐activated Cl^−^ channels (VACC): these are activated when osmolality in extracellular environments decreases or intracellular osmolality increases, leading to swelling of the cells. As the osmolality in our extracellular and intracellular solution was within the normal range (292–295 mmols) cell volume should not alter significantly: in addition VACC have a typical permeability order of anions: I^−^>Br^−^>Cl^−^>F^−^>gluconate, which was different from that seen in our study (I^−^>Cl^−^>Br^−^ >Aspartate^−^). Furthermore, transcripts for VACC are expressed at much lower levels than CLIC1 in hBECs as observed in our RNA Seq datasets.

The whole Cl^−^ cell currents induced by isoproterenol and forskolin in the present study shared some features of the outwardly rectifying Cl^−^ channels (ORCC) studied in Calu‐3 and in a previous study using hBECs (Szkotak et al. 2003). Both currents showed a typical property of outward rectification and activation by intracellular cAMP. However, ORCC showed a typical ion permeability of I^−^>Br^−^≫Cl^−^ which was different from that in the present study. ORCC are sensitive to 4,4′‐diisothiocyanatostilbene‐2, 2′‐disulfonic acid, and to 9‐AC, but not to 4,4′‐dinitrostilbene‐2,2′‐disulfonic acid (DNDS), while the currents induced here are sensitive to DIDs, but not to NPPB (Schwiebert et al. 1994).

In whole‐cell patch clamp studies, many researchers have demonstrated that hBECs express cAMP‐activated whole‐cells currents which have properties characteristic of CFTR‐mediated currents with 8‐ to 10‐ps single conductance and a linear current–voltage relationship that was blocked by carboxylic acid (DPC) but insensitive to DIDS. In the present study, we found that one of the more potent and specific CFTR antagonists available, PPQ‐102, almost completely abolished the whole‐cell currents induced by isoproterenol or forskolin. However, the lack of CFTR transcript in our RNA Seq datasets mean that it is unlikely (although still possible) that CFTR contributes to the whole‐cell currents induced by increased cAMP in the present study.

A major problem with studying Cl^−^ channels is the lack of specificity of the available inhibitors. While the antagonists used in this study have been partially profiled against Cl^−^ channel family members, no previous studies have described comprehensively the effect of these inhibitors on CLIC family members, and hence by themselves, the inhibitor studies we performed are somewhat inconclusive. We therefore chose to utilize a siRNA knockdown approach to attempt to tease out the contribution of CLIC1 to Cl^−^ currents in hBECs. While siRNA knockdown can affect off‐target genes, when these assays are carefully designed and optimized and validated, it has been proven to be an accurate and robust method to study specific genes. These studies show that knockdown of CLIC1 inhibits a substantial portion of the cAMP‐induced Cl^−^ current in these cells, suggesting a direct or indirect contribution of CLIC1 to this current.

In conclusion, we demonstrate here the expression of a number of CLIC family members in hBECs, with CLIC1 being the most abundant at the mRNA level. Our data suggest it is possible that manipulating CLIC1 could be a way to further enhance Cl^−^ channel activity and that this could therefore prove a novel therapeutic approach to aid in the treatment of conditions where chloride homeostasis is impacted such as cystic fibrosis.

## Conflict of interest

None declared.

## Data Accessibility

## Supporting information




**Table S1:** Abundance of Chloride channel (1a) and CLIC family (1b) RNA transcripts in human bronchial epithelial cells as determined using RNA Seq.

## References

[phy213508-bib-0001] Anderson, M. P. , and M. J. Welsh . 1991 Calcium and cAMP activate different chloride channels in the apical membrane of normal and cystic fibrosis epithelia. Proc. Natl. Acad. Sci. USA 88:6003–6007. 1712478 10.1073/pnas.88.14.6003 PMC52010

[phy213508-bib-0002] Averaimo, S. , R. H. Milton , M. R. Duchen , and M. Mazzanti . 2010 Chloride intracellular channel 1 (CLIC1): sensor and effector during oxidative stress. FEBS Lett. 584:2076–2084. 20385134 10.1016/j.febslet.2010.02.073

[phy213508-bib-0003] Cliff, W. H. , and R. A. Frizzell . 1990 Separate Cl‐ conductances activated by cAMP and Ca2 + in Cl(‐)‐secreting epithelial cells. Proc. Natl. Acad. Sci. USA 87:4956–4960. 2164213 10.1073/pnas.87.13.4956 PMC54240

[phy213508-bib-0004] Edwards, J. C. 2006 The CLIC1 chloride channel is regulated by the cystic fibrosis transmembrane conductance regulator when expressed in *Xenopus oocytes* . J. Membr. Biol. 213:39–46. 17347778 10.1007/s00232-006-0059-5 PMC2665869

[phy213508-bib-0005] Edwards, J. C. , and C. R. Kahl . 2010 Chloride channels of intracellular membranes. FEBS Lett. 584:2102–2111. 20100480 10.1016/j.febslet.2010.01.037 PMC2929963

[phy213508-bib-0006] Haq, I. J. , M. A. Gray , J. P. Garnett , C. Ward , and M. Brodlie . 2016 Airway surface liquid homeostasis in cystic fibrosis: pathophysiology and therapeutic targets. Thorax 71:284–287. 26719229 10.1136/thoraxjnl-2015-207588

[phy213508-bib-0007] Kheirallah, A. K. , C. H. de Moor , A. Faiz , I. Sayers , and I. P. Hall . 2017 Lung function associated gene Integrator Complex subunit 12 regulates protein synthesis pathways. BMC Genom. 18:248. 10.1186/s12864-017-3628-3 PMC5364626 28335732

[phy213508-bib-0008] Malekova, L. , J. Tomaskova , M. Novakova , P. Stefanik , J. Kopacek , B. Lakatos , et al. 2007 Inhibitory effect of DIDS, NPPB, and phloretin on intracellular chloride channels. Pflugers Arch. 455:349–357. 17611769 10.1007/s00424-007-0300-9

[phy213508-bib-0009] Peretti, M. , M. Angelini , N. Savalli , T. Florio , S. H. Yuspa , and M. Mazzanti . 1848 Chloride channels in cancer: focus on chloride intracellular channel 1 and 4 (CLIC1 AND CLIC4) proteins in tumor development and as novel therapeutic targets. Biochim. Biophys. Acta 2523–2531:2015. 10.1016/j.bbamem.2014.12.012 25546839

[phy213508-bib-0010] Qiu, M. R. , L. Jiang , K. I. Matthaei , S. M. Schoenwaelder , T. Kuffner , P. Mangin , et al. 2010 Generation and characterization of mice with null mutation of the chloride intracellular channel 1 gene. Genesis 48:127–136. 20049953 10.1002/dvg.20590

[phy213508-bib-0011] Salao, K. , L. Jiang , H. Li , V. W. Tsai , Y. Husaini , P. M. Curmi , et al. 2016 CLIC1 regulates dendritic cell antigen processing and presentation by modulating phagosome acidification and proteolysis. Biol. Open 5:620–630. 27113959 10.1242/bio.018119 PMC4874360

[phy213508-bib-0012] Schwiebert, E. M. , T. Flotte , G. R. Cutting , and W. B. Guggino . 1994 Both CFTR and outwardly rectifying chloride channels contribute to cAMP‐stimulated whole cell chloride currents. Am. J. Physiol. 266:C1464–C1477. 7515570 10.1152/ajpcell.1994.266.5.C1464

[phy213508-bib-0013] Schwiebert, E. M. , M. E. Egan , T. H. Hwang , S. B. Fulmer , S. S. Allen , G. R. Cutting , et al. 1995 CFTR regulates outwardly rectifying chloride channels through an autocrine mechanism involving ATP. Cell 81:1063–1073. 7541313 10.1016/s0092-8674(05)80011-x

[phy213508-bib-0014] Szkotak, A. J. , S. F. Man , and M. Duszyk . 2003 The role of the basolateral outwardly rectifying chloride channel in human airway epithelial anion secretion. Am. J. Respir. Cell Mol. Biol. 29:710–720. 12777250 10.1165/rcmb.2003-0109OC

[phy213508-bib-0015] Tonini, R. , A. Ferroni , S. M. Valenzuela , K. Warton , T. J. Campbell , S. N. Breit , et al. 2000 Functional characterization of the NCC27 nuclear protein in stable transfected CHO‐K1 cells. FASEB J. 14:1171–1178. 10834939 10.1096/fasebj.14.9.1171

[phy213508-bib-0016] Tulk, B. M. , S. Kapadia , and J. C. Edwards . 2002 CLIC1 inserts from the aqueous phase into phospholipid membranes, where it functions as an anion channel. Am. J. Physiol. Cell Physiol. 282:C1103–C1112. 11940526 10.1152/ajpcell.00402.2001

[phy213508-bib-0017] Ulmasov, B. , J. Bruno , P. G. Woost , and J. C. Edwards . 2007 Tissue and subcellular distribution of CLIC1. BMC Cell Biol. 8:8. 17326840 10.1186/1471-2121-8-8 PMC1820597

[phy213508-bib-0018] Wadsworth, S. J. , H. S. Nijmeh , and I. P. Hall . 2006 Glucocorticoids increase repair potential in a novel in vitro human airway epithelial wounding model. J. Clin. Immunol. 26:376–387. 16786432 10.1007/s10875-006-9029-z

[phy213508-bib-0019] Wang, X. , J. Venable , P. LaPointe , D. M. Hutt , A. V. Koulov , J. Coppinger , et al. 2006 Hsp90 cochaperone Aha1 downregulation rescues misfolding of CFTR in cystic fibrosis. Cell 127:803–815. 17110338 10.1016/j.cell.2006.09.043

[phy213508-bib-0020] Warton, K. , R. Tonini , W. D. Fairlie , J. M. Matthews , S. M. Valenzuela , M. R. Qiu , et al. 2002 Recombinant CLIC1 (NCC27) assembles in lipid bilayers via a pH‐dependent two‐state process to form chloride ion channels with identical characteristics to those observed in Chinese hamster ovary cells expressing CLIC1. J. Biol. Chem. 277:26003–26011. 11978800 10.1074/jbc.M203666200

