# Supplementary material for: Chloride intracellular channel 1 (CLIC1) contributes to modulation of cyclic AMP‐activated whole‐cell chloride currents in human bronchial epithelial cells
Source: Physiol Rep. 2018 Jan 25;6(2):e13508. doi: 10.14814/phy2.13508 (PMC5789713; doi:10.14814/phy2.13508)
Supplement: Supplementary file 1 — Table S1: Abundance of Chloride channel (1a) and CLIC family (1b) RNA transcripts in human bronchial epithelial cells as determined using RNA Seq. [file PHY2-6-e13508-s001.pdf]

Table 1a

Chloride channels	Number of channels in family	Percent Expression
P64 gene family (CLIC) chloride channel:	6	61.28
Volume regulated anion channel:	5	18.27
Calcium-activated chloride channel:	19	11.74
ClC-family:	9	5.12
Maxi chloride channel:	3	3.6
Ligand gated (GABA) chloride channel:	7	0
CFTR:	1	0

Table 1b

P64 gene family (CLIC) chloride channel:	Percent Expression
CLIC1	80.37
CLIC4	18.72
CLIC3	0.9
CLIC6	0.01
CLIC2	0
CLIC5	0
